# Editorial: Integration of Hormonal Signals Shaping Root Growth, Development, and Architecture

**DOI:** 10.3389/fpls.2021.634066

**Published:** 2021-02-12

**Authors:** Javier Agusti, Eswarayya Ramireddy, Javier Brumos

**Affiliations:** ^1^Institute of Molecular and Cellular Biology of Plants (IBMCP), Consejo Superior de Investigaciones Científicas (CSIC), Universitat Politécnica de Valéncia, Valencia, Spain; ^2^Biology Division, Indian Institute of Science Education and Research, Tirupati, India; ^3^Program in Genetics, Department of Plant and Microbial Biology, North Carolina State University, Raleigh, NC, United States

**Keywords:** development, root growth, plant hormones, regulation, crops, stress, auxin, ethylene

Plants need to constantly modify their growth and development to adapt to the ever-changing conditions and thus optimize the use of available resources. Over millions of years of evolution, plants have developed sophisticated mechanisms that allow them to integrate the information from external stimuli with their own internal programs to generate appropriate developmental outputs. Plant hormones act as signals in this integration process and contribute to the extraordinary plant morphological plasticity. The root system is a key determinant of plant development, exploring uncharted territory in the soil and serving as an interface between plant and rhizosphere. The roots enable the selective uptake of water and nutrients whereas exclude phytotoxic compounds. All these functions are maximized by the root morphological plasticity. The root anatomy is defined by regular patterns originated by cell division in the root apical meristem and consecutive cell differentiation that result in the different tissues composing the root. Due to these remarkable regular patterns, the root is an exceptionally useful system to study the effects of hormones on growth and development. Changes in plant hormone homeostasis via synthesis, modification, catabolism, and transport as well as the modulation of signaling components are key for a tunable and resettable hormone response system that controls root growth and development. Additional layers of fine-tuning are achieved by the intense cross-talk between different plant hormones at various levels. Phytohormones affect every stage of plant development including agriculturally important processes. Therefore, understanding how particular hormones and gene expression networks interact to coordinate root growth and development in a dynamic environment is essential, not only for developmental biology but also for the design of the next generation of crops coping with current agricultural challenges such as increasing food demand and climate change.

The main objective of this Research Topic is to gather current works studying the integration of hormonal signals in the root, a key question in plant developmental biology. If we improve our understanding on how hormones coordinate root growth, development, and architecture we can then improve our crops.

In crops, especially those cropped intensively such as cereals, developing an optimal root architecture is crucial for yield enhancement and space optimization without disturbing proper nutrient absorption. Therefore, the original research done by Sun et al., “A strigolactone signal inhibits secondary lateral root development in rice” aims to understand the hormonal control of secondary lateral roots in rice. Based on previous knowledge indicating that strigolactone signaling regulates lateral root development, the authors investigated whether secondary lateral roots development is also regulated by strigolactone and what the relationship between strigolactone and auxin signaling could be. Following an approach combining genetic analyses and pharmacological applications the authors show that while auxin applications on rice mutants deficient in strigolactone biosynthesis and sensitivity increased the number of secondary lateral roots, adding strigolactone treatments to the experiment only reduced the auxin effect in strigolactone biosynthesis mutants, but not in strigolactone-insensitive mutants. Thus, Sun et al. work points out that auxin and strigolactone have opposing effects in the process, and that the regulation of their action is complex.

Another original research article in this Topic (Liang et al.) focuses on how the crop load influences growth and hormone changes in the roots of “Red Fuji” apple. Crop load represents a crucial factor for the shoot and root growth and development of apple trees. The authors analyze the effects of an extensive range of crop loads on hormone levels and growth in apple roots. Higher crop loads resulted in lower root growth and non-fruiting plants exhibit elevated root growth. During the root growth peaks, the levels of cytokinins, indole acetic acid, and gibberellic acid were also at their highest. Together with the increase of crop load, the hormone levels were gradually decreasing within each peak. The results of this work suggest that root growth is positively correlated with hormone levels during the fruit growth phase, and that the reduction in root growth caused by crop load might be regulated by the reduction on hormone levels.

A collection of reviews bringing together different levels of root development regulation is also present in this Research Topic.

Ramachandran et al. focus their review on how water limitations modulate xylem development. In normal conditions, correct xylem development depends on the balance between hormones such as auxin, cytokinin, brassinosteroids, jasmonic acid, and abscisic acid. Drought alters that balance leading to a reduction of cambial cellular proliferation accompanied by an increase of xylem vessels production. These vessels, however, are significantly thinner, enhancing water transport efficiency while protecting the plant from embolism. This developmental plasticity is key for acclimation to drought. However, to date, little is known about the molecular mechanisms underlying it. Data previously produced by the authors' lab indicate that abscisic acid is, most likely, at the center of this plastic response to drought. The authors elegantly explain why breeding for xylem developmental alterations emulating those induced by drought may improve agricultural systems sustainability by enhancing water usage efficiency. Finally, Ramachandran et al. also point out that a number of links between environmental factors and the molecular regulation of xylem development are still missing and that implementing new technological approaches such as single cell sequencing, sheet fluorescence microscopy combined with Growth and Luminiscence Observatory for roots (GLO-Roots) or natural variation analyses to the study of xylem development hold great promise in identifying them.

The review by Xu et al., hones in the integration of jasmonic acid and ethylene into auxin signaling and how these interactions coordinate root development through the activity of ERF and HD-ZIP transcription factor families. *ERF109* is induced by jasmonic acid. ERF109 induces local auxin production in the root stimulating lateral root formation. Recently, *ERF109* has also been associated with plant regeneration. Ethylene induces the expression of *ERF1* which activates auxin biosynthesis and transport that in turn inhibits the elongation of the primary root. Ethylene also upregulates the expression of *HB52*, a member of the HD-ZIP transcription factor family. HB52 regulates auxin transport, altering auxin homeostasis in roots and inhibiting primary root elongation. The examples discussed in this review represent key crosstalk nodes between ethylene, jasmonic acid, and auxin that regulate root development.

Cell fate determination and stem cells maintenance at the root apical meristem (RAM) are key for proper growth and organogenesis of the primary root. Many intrinsic gene regulatory networks that involve phytohormones, peptide signaling, microRNAs and transcription factors are integrating the information from the environmental cues to maintain the characteristics of the root stem cell niche. Recently, studies indicate the cellular redox status and the presence of ROS can also play a pivotal role in this complex process. In the current Research Topic, the work of Zhou et al. portraits the panoramic view of ROS, as a fine-tuner of plant stem cell proliferation and differentiation. Zhou et al. summarize the recent findings in the field of ROS and the role of ROS in root development with an emphasis on root stem cell differentiation and maintenance, root hair differentiation and during the cell-cycle progression. Further, Zhou et al. not only describe the crosstalk between known genetic factors, hormones, and peptides with ROS, but also its newly found role in aerenchyma formation.

Calleja-Cabrera et al. discuss the root growth adaptation to climate change in crops. Climate change is the biggest threat to crop productivity in the world. High temperatures caused by global warming have deleterious effects on plant growth and development. As mentioned above, roots are essential for water and nutrient uptake. Modifications of soil temperatures affect root growth restraining production. Root architecture is affected by warmer soils. Increasing temperatures trigger diverse physiological and metabolic responses in the plant coping with warmer soils. Different regulatory mechanisms control the plant responses that prevent root cell damage and root growth impairment. Increasing temperatures are accompanied by other abiotic and biotic stresses such as drought, salinity, nutrient deficiencies, and pathogen infections that affect hormone homeostasis and gene expression. The authors not only discuss the latest studies in the field but also future research paths.

In conclusion, these original and review articles discuss the large interest and substantial progress that has been made in the field of root biology, especially on shedding light on hormonal cross-talk in root development.

## Author Contributions

JB prepared the outline of the manuscript. All authors wrote and improved parts of the manuscript.

## Conflict of Interest

The authors declare that the research was conducted in the absence of any commercial or financial relationships that could be construed as a potential conflict of interest.

